# Identification of Potential Diagnostic Biomarkers From Circulating Cells During the Course of Sleep Deprivation-Related Myocardial Infarction Based on Bioinformatics Analyses

**DOI:** 10.3389/fcvm.2022.843426

**Published:** 2022-03-17

**Authors:** Xiang Chen, Qian Li, Zhong Zhang, Minjing Yang, E. Wang

**Affiliations:** ^1^Department of Anesthesiology, Xiangya Hospital Central South University, Changsha, China; ^2^National Clinical Research Center for Geriatric Disorders (Xiangya Hospital), Xiangya Hospital Central South University, Changsha, China

**Keywords:** myocardial infarction, sleep deprivation, diagnostic biomarker, immune cells, bioinformatic analysis

## Abstract

**Background:**

Myocardial infarction (MI) is the leading cause of death from non-infectious diseases worldwide and results in rapid deterioration due to the sudden rupture of plaques associated with atherosclerosis, a chronic inflammatory disease. Sleep is a key factor that regulates immune homeostasis of the body. The imbalance in circulating immune cells caused by sleep deprivation (SD) may represent a risk factor leading to the rapid deterioration of plaques and MI. Therefore, it is of profound significance to identify diagnostic biomarkers for preventing SD-related MI.

**Methods:**

In the present study, we identified coexpressed differentially expressed genes (co-DEGs) between peripheral blood mononuclear cells from MI and SD samples (compared to controls) from a public database. LASSO regression analysis was applied to identify significant diagnostic biomarkers from co-DEGs. Moreover, receiver operating characteristic (ROC) curve analysis was performed to test biomarker accuracy and diagnostic ability. We further analyzed immune cell enrichment in MI and SD samples using the CIBERSORT algorithm, and the correlation between biomarkers and immune cell composition was assessed. We also investigated whether diagnostic biomarkers are involved in immune cell signaling pathways in SD-related MI processes.

**Results:**

A total of 10 downregulated co-DEGs from the sets of MI-DEGs and SD-DEGs were overlapped. After applying LASSO regression analysis, SYTL2, KLRD1, and C12orf75 were selected and validated as diagnostic biomarkers using ROC analysis. Next, we found that resting NK cells were downregulated in both the MI samples and SD samples, which is similar to the changes noted for SYTL2. Importantly, SYTL2 was strongly positively correlated not only with resting NK cells but also with most genes related to NK cell markers in the MI and SD datasets. Moreover, SYTL2 was highly associated with genes in NK cell signaling pathways, including the MAPK signaling pathway, cytotoxic granule movement and exocytosis, and NK cell activation. Furthermore, GSEA and KEGG analyses provided evidence that the DEGs identified from MI samples with low vs. high SYTL2 expression exhibited a strong association with the regulation of the immune response and NK cell-mediated cytotoxicity.

**Conclusion:**

In conclusion, SYTL2, KLRD1, and C12orf75 represent potential diagnostic biomarkers of MI. The association between SYTL2 and resting NK cells may be critically involved in SD-related MI development and occurrence.

## Introduction

Myocardial infarction (MI) has become one of the major causes of death and disability worldwide (1, 2). MI mainly occurs in patients with coronary artery disease (CAD), especially coronary atherosclerosis, who experience unstable periods with activated inflammation in the vascular wall (2, 3). Although the exact cause of MI remains unknown, traditional risk factors, including hypertension, smoking, diabetes, obesity and unhealthy diet, might greatly increase the incidence of MI. In addition, up to 5% of elderly people (>75 years old) develop silent MI with no history of established heart disease (4). Once MI occurs, heart failure, heart attack, and cardiac arrest might follow if not treated in a timely and effective manner, ultimately leading to death. Many epidemiological studies and randomized controlled clinical trials have suggested that promoting a healthy lifestyle and diet can help manage hypertriglyceridemia for the prevention of atherosclerotic cardiovascular disease and MI (5, 6).

A short duration of sleep and sleep deprivation (SD) show secular trends alongside changes in modern society that require longer hours of work, which has been considered a global health epidemic (7, 8). Studies from many countries have indicated that SD is correlated with overall health and mortality as well as specific cardiovascular and/or metabolic disorders (9). In a prospective observational study including 461,347 participants free of relevant cardiovascular disease, the researchers found that cases with habitual self-reported short (<6 h) sleep duration had a 20% increased multivariable-adjusted risk of incident for MI compared to cases who sleep 6–9 h/night (7). Healthy sleep duration mitigated MI risk even among individuals at high genetic risk (7). SD contributes to a greater risk of MI, which might result in metabolic and endocrine dysfunction, an imbalanced immune system, and endothelial dysfunction caused by the lack of sleep (10–13). Given the global burden of MI, it is vital to identify novel molecular biomarkers involved in the mechanism of SD-related MI for early detection and continuous monitoring to guide health care professionals, which might help to ensure formulation of the correct therapeutic regimen.

With the remarkable evolution of bioinformatics, microarray gene expression data can be used to identify hub genes and differentially involved signaling pathways in the course of MI, which promotes a comprehensive perspective on key cellular and molecular mechanisms. Research based on bioinformatics methods found that IL1R2, IRAK3, and THBD expression levels were notably higher in peripheral blood mononuclear cells (PBMCs) of patients with acute MI (AMI) and were identified as diagnostic markers of AMI (14). These genes were also significantly associated with various subtypes of immune cells within the AMI samples (14). The occurrence of MI is accompanied by composition changes in T cells and natural killer cells (NK cells) as well as monocyte and macrophage infiltration (15). Of note, long-term SD leads to elevated markers of inflammatory activity and an abnormal number of immune cells, which are in the same range as that observed in individuals at risk for developing cardiovascular disease in the future (16). For the mentioned reasons, evaluating and ascertaining the distinctions within the proportion of immune cells are important in clarifying the potential mechanisms of SD-related MI.

The present study obtained PBMC whole-genome microarray datasets from a public database to identify co-expressed differentially expressed genes (co-DEGs) within SD and MI samples. Then, least absolute shrinkage and selection operator (LASSO) regression analysis was applied to screen and identify diagnostic biomarkers of MI based on the co-DEGs. Next, the correlation between diagnostic markers and the composition of immune cells was analyzed using CIBERSORT algorithms. Gene set enrichment analysis (GSEA) and Kyoto Encyclopedia of Genes and Genomes (KEGG) analysis were performed to increase our understanding of the underlying immune mechanisms involved in the development of MI.

## Methods

### Microarray Data

Two peripheral-blood whole-genome microarray datasets related to MI, GSE59867 and GSE62646, were selected and obtained from the Gene Expression Omnibus (GEO) database (https://www.ncbi.nlm.nih.gov/GEO/). Both datasets were based on the GPL6244 platform of Affymetrix Human Gene 1.0 ST Array. The GSE59867 dataset contains data from PBMC samples from patients (*n* = 111) with ST-segment elevation myocardial infarction (STEMI) and stable CAD patients (*n* = 46) without a history of MI. The expression profiles of 28 patients with STEMI and 14 stable CAD patients without a history of MI were included in the GSE62646 dataset. These two datasets were combined by batch correction with the “combat” function of the “sva” package of R using empirical Bayes frameworks (17), and then used to identify the co-DEGs of MI. The GSE48060 dataset was obtained from the PBMC samples of 31 patients with MI and 21 controls based on the GPL570 platform of Affymetrix Human Genome U133 Plus 2.0 Array and were downloaded as a validation dataset for the co-DEGs of MI. To identify the DEGs of SD, the GSE37667 dataset based on the GPL570 platform of Affymetrix Human Genome U133 Plus 2.0 Array was downloaded. PBMC gene expression profiles of nine healthy male volunteers at baseline at night and after 60 h of prolonged SD were collected.

Gene symbols were transformed from the probes in each dataset based on their probe annotation files. The final expression value of the gene corresponding to multiple probes was obtained by calculating the average expression value. Then, we applied background correction and quartile normalization for all gene expression values to obtain normally distributed expression values using the “limma” package in R.

### Screening of DEGs

DEGs between MI and CAD samples as well as SD and control samples were identified using Wilcoxon test *via* the “limma” package in R. Because alterations of RNA expression levels in PBMCs are generally lower than that in other human tissues (18), we set the fold change (FC) > 1.3 and original *P* < 0.05 as significance cut-offs to screen the DEGs based on the recommended methods from studies that designed and uploaded the GSE59867 and GSE62646 datasets (19–21). Volcano plots were used to visualize the distribution of the DEGs. Co-DEGs between MI-DEGs (GSE59867 and GSE62646 datasets) and SD-DEGs (GSE37667 dataset) were identified as the genes overlapping in these three gene sets and were visualized using a Venn diagram.

### Identification of Diagnostic Biomarkers

To identify significant diagnostic biomarkers for the discrimination of MI and CAD cases, we performed LASSO regression analysis using the “glmnet” package in R. As a type of shrinkage method for linear regression models, LASSO regression analysis identifies the subset of predictors from the best fitting model by k-fold cross validation, which effectively reduces the prediction error. With a constraint imposed on the model parameters, the shrinkage process is conducted to shrink the regression coefficients of some variables toward zero. Variables with a regression coefficient unequal to zero are included in the final model. Thus, the risk score of each case was calculated using the following formula: Risk score = Σexpgenei^*^ βi, where expgenei represents the gene expression value, and βi represents the regression coefficient of gene i extracted from the LASSO regression analysis for the GSE59867 dataset.

### Evaluation of Diagnostic Value of MI Biomarkers

To evaluate the accuracy and diagnostic ability of the biomarkers and risk score, receiver operating characteristic (ROC) curve analysis was applied for the GSE59867 dataset using the “pROC” package in R. In addition, we also used the GSE62646 and GSE48060 datasets as external validation datasets to verify the diagnostic value of the identified biomarkers. The area under the curve (AUC) of the ROC curve was calculated with sensitivity and specificity values and visualized using the “pROC” package in R.

### Discovery of Immune Cell Subtypes

To quantify the relative population-specific immune cell enrichment for each sample, the CIBERSORT algorithm was performed with 1,000 permutations to calculate the normalized enrichment scores of 22 types of immune cells using the “cibersort” package in R (22). The CIBERSORT algorithm improves deconvolution performance to obtain normalized enrichment scores based on support vector regression, which is a machine learning approach. The CIBERSORT gene signature matrix, termed LM22, contains 547 genes and distinguishes 22 types of immune cell subtypes. Therefore, the enrichment scores can be inferred from the eigenmatrix, such as LM22, and gene expression in each sample within a given dataset. In addition, we further classified the 22 immune cell types into 4 aggregated immune cell types, including total lymphocytes, total dendritic cells (sum of activated and resting dendritic cell percentages), total macrophages (sum of M0, M1, and M2 macrophage percentages) and total mast cells (sum of activated and resting mast cell percentages) (23). We used the two-sided Wilcoxon test to compare differences in the composition of 22 immune cell subtypes between two groups (MI vs. CAD, SD vs. control) and visualized the results with violin plots using the “vioplot” package in R. Correlation analysis of 22 immune cell subtypes was visualized using the “corrplot” package.

### Correlation Analysis Between Diagnostic Biomarkers and Immune Cell Subtypes

The correlation of the diagnostic biomarkers with the differentially distributed immune cell subtypes was analyzed among the GSE59867, GSE62646, and GSE37667 datasets. To further explore the correlation with specific immune cell markers, we downloaded gene sets related to specific human immune cell markers from the CellMarker database (http://biocc.hrbmu.edu.cn/CellMarker/) and performed Pearson correlation analysis between diagnostic biomarkers and each gene for immune cell markers.

### Functional Enrichment Analysis of DEGs

To explore potential biological functions and significant signaling pathways of DEGs associated with the diagnostic biomarker, we performed Gene Ontology (GO) and KEGG pathway enrichment analyses (24) based on the DAVID tools (http://david.ncifcrf.gov/) and visualized the results using the “clusterProfiler” package in R. The strict cut-off of a false discovery rate (FDR) < 0.05 and adjusted *P*-value < 0.05 was used to identify statistically significant GO terms. In addition, GSEA was also conducted to explore the functional terms correlated to diagnostic biomarkers based on an NOM *P*-value < 0.05 and NES > 1.

### Statistical Analysis

All statistical analyses were conducted using R software (Version 4.0.2; R Foundation for Statistical Computing, Vienna, Austria). Two groups of boxplots for continuous variables were analyzed using the Wilcoxon test. ROC curve analysis was conducted to evaluate the diagnostic efficacy of diagnostic biomarkers for MI. All statistical tests were two-sided, and a *P-*value < 0.05 was considered statistically significant.

## Results

### Identification of DEGs

**Figure 1:**
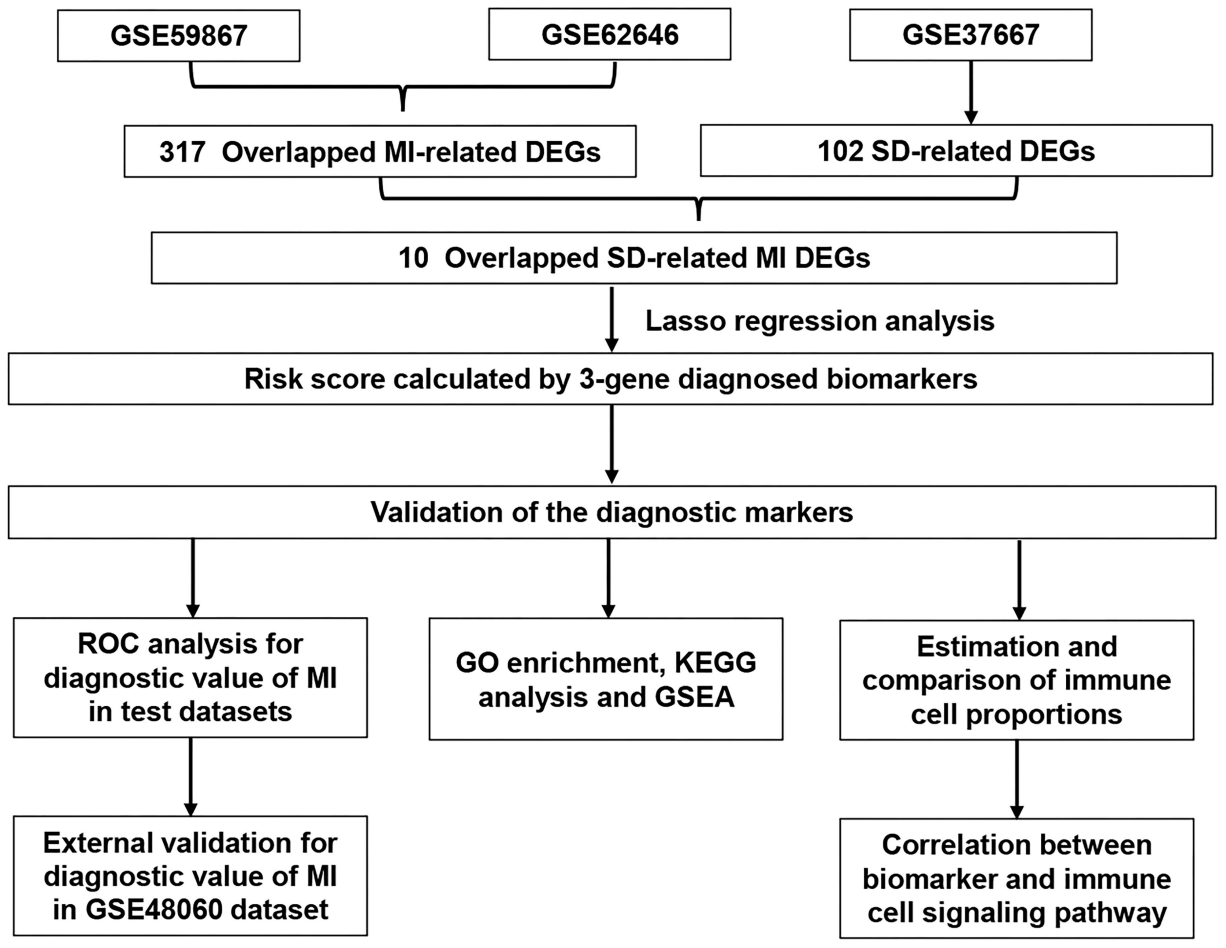
Flowchart describing the process used to identify and validate the diagnostic biomarkers of myocardial infarction. DEGs, differentially expressed genes; MI, myocardial infarction; SD, sleep deprivation; ROC, receiver operating characteristic.

**Figure 2:**
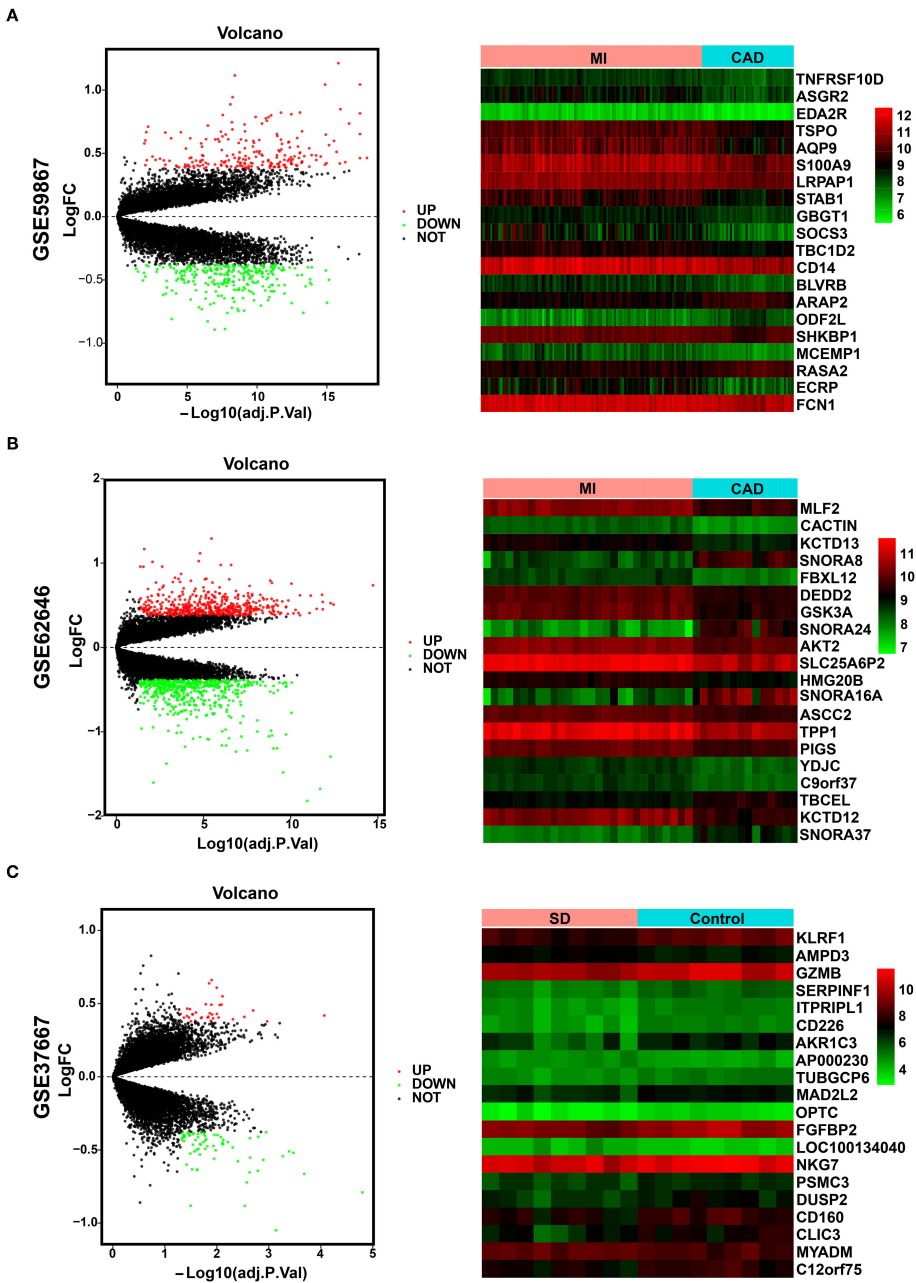
Identification of MI-DEGs and SD-DEGs. **(A)** Volcano plot (left) and heatmap (right) of the GSE59867 dataset. In total, 491 MI-DEGs were identified from the GSE59867 dataset between the MI and CAD PBMC samples, including 282 downregulated and 209 upregulated genes. **(B)** Volcano plot (left) and heatmap (right) of the GSE62646 dataset. In total, 1,108 MI-DEGs were identified from the GSE62646 dataset between the MI and CAD PBMC samples, including 593 downregulated and 515 upregulated genes. **(C)** Volcano plot (left) and heatmap (right) of the GSE37667 dataset. In total, 102 SD-DEGs were identified from the GSE37667 dataset between the SD and control PBMC samples, including 68 downregulated and 34 upregulated genes. DEGs, differentially expressed genes; MI, myocardial infarction; SD, sleep deprivation; CAD, coronary artery disease; PBMCs, peripheral blood mononuclear cells.

The overall data processing workflow is presented in Figure 1. For DEGs between the MI and CAD samples, 491 DEGs were screened from the GSE59867 dataset, including 282 downregulated and 209 upregulated genes (Figure 2A and Supplementary Table 1). A total of 1,108 DEGs were identified from the GSE62646 dataset, including 593 downregulated and 515 upregulated genes (Figure 2B and Supplementary Table 1). In addition, a total of 102 DEGs between the SD and control samples were obtained from the GSE37667 dataset, including 68 downregulated genes and 34 upregulated genes (Figure 2C and Supplementary Table 1). Volcano plots were used to visualize the distribution of the DEGs (Figure 2). Hierarchical clustering analysis demonstrated differences in the expression patterns of the top 20 MI-DEGs and SD-DEGs between the two groups (Figure 2).

### Identification of Diagnostic Biomarkers

**Figure 3:**
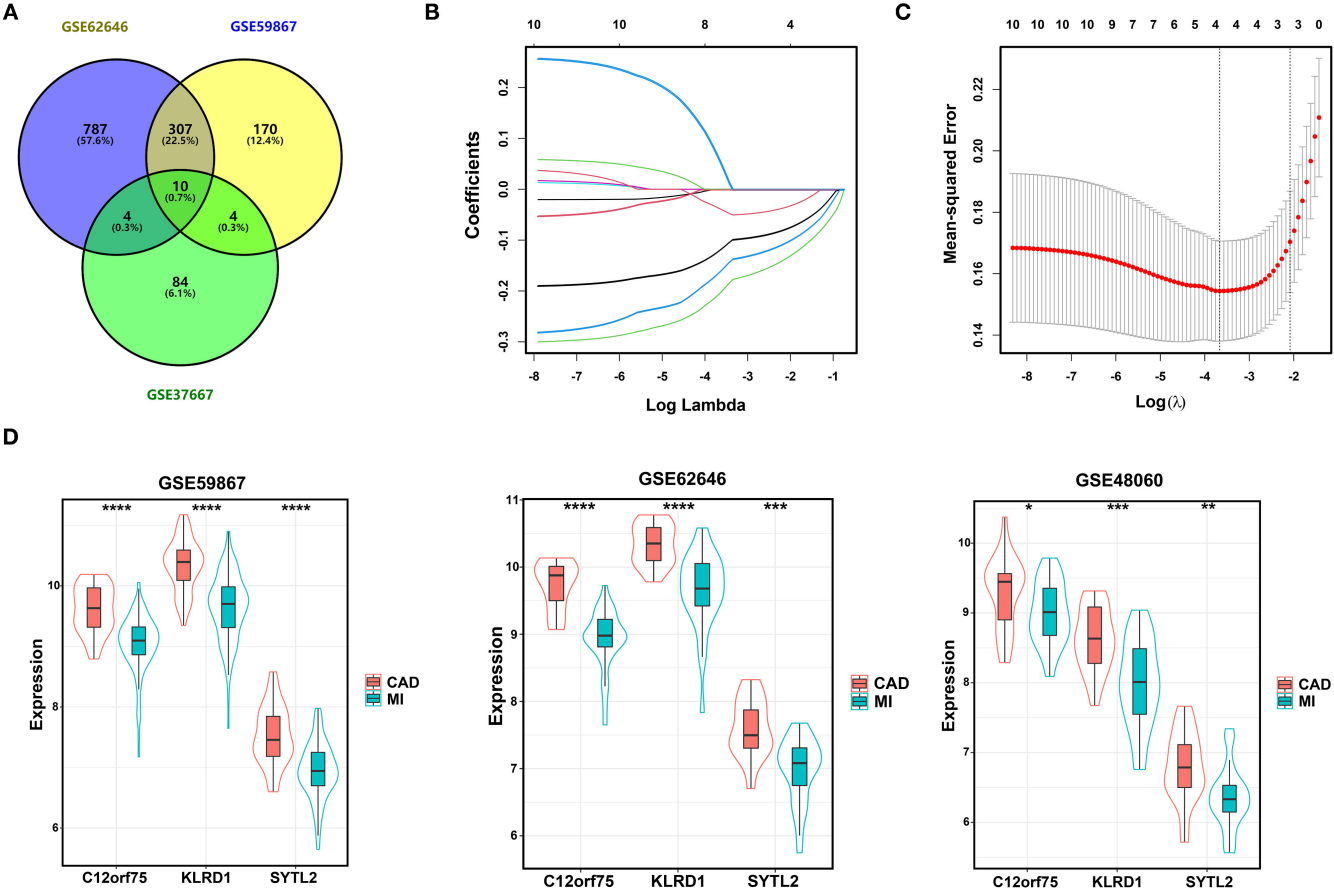
Identification of diagnostic biomarkers for SD-related MI. **(A)** Venn diagram of two sets of MI-DEGs and a set of SD-DEGs. **(B,C)** LASSO coefficient profiles and LASSO deviance profiles. **(D)** The expression levels of three biomarkers identified by LASSO regression analysis between MI and CAD samples in the GSE59867, GSE62646, and GSE48060 datasets. *P < 0.05; **P < 0.01; ***P < 0.001; ****P < 0.0001. MI, myocardial infarction; SD, sleep deprivation; CAD, coronary artery disease.

We integrated the two sets of MI-DEGs and one set of SD-DEGs using a Venn diagram, as shown in Figure 3A. A total of 10 genes overlapping among the three datasets were identified as co-DEGs, all of which were downregulated in both the MI-DEGs and SD-DEGs. Next, we performed LASSO regression analysis to identify the diagnostic biomarkers for MI in the GSE59867 dataset (Figures 3B,C). After running cross-validation likelihood 1,000 times, a subset of three biomarkers from the co-DEGs was determined: Synaptotagmin Like 2 (SYTL2), Killer Cell Lectin Like Receptor D1 (KLRD1), and Chromosome 12 Open Reading Frame 75 (C12orf75). To validate the different expression levels of the three diagnostic biomarkers between MI and CAD samples, we analyzed the different expression levels of the three diagnostic biomarkers between MI and CAD PBMC samples in the three datasets and found that the expression levels of all of these genes were notably lower in MI samples compared with CAD samples (all *P* < 0.05) (Figure 3D).

### Validation of Diagnostic Biomarkers

**Figure 4:**
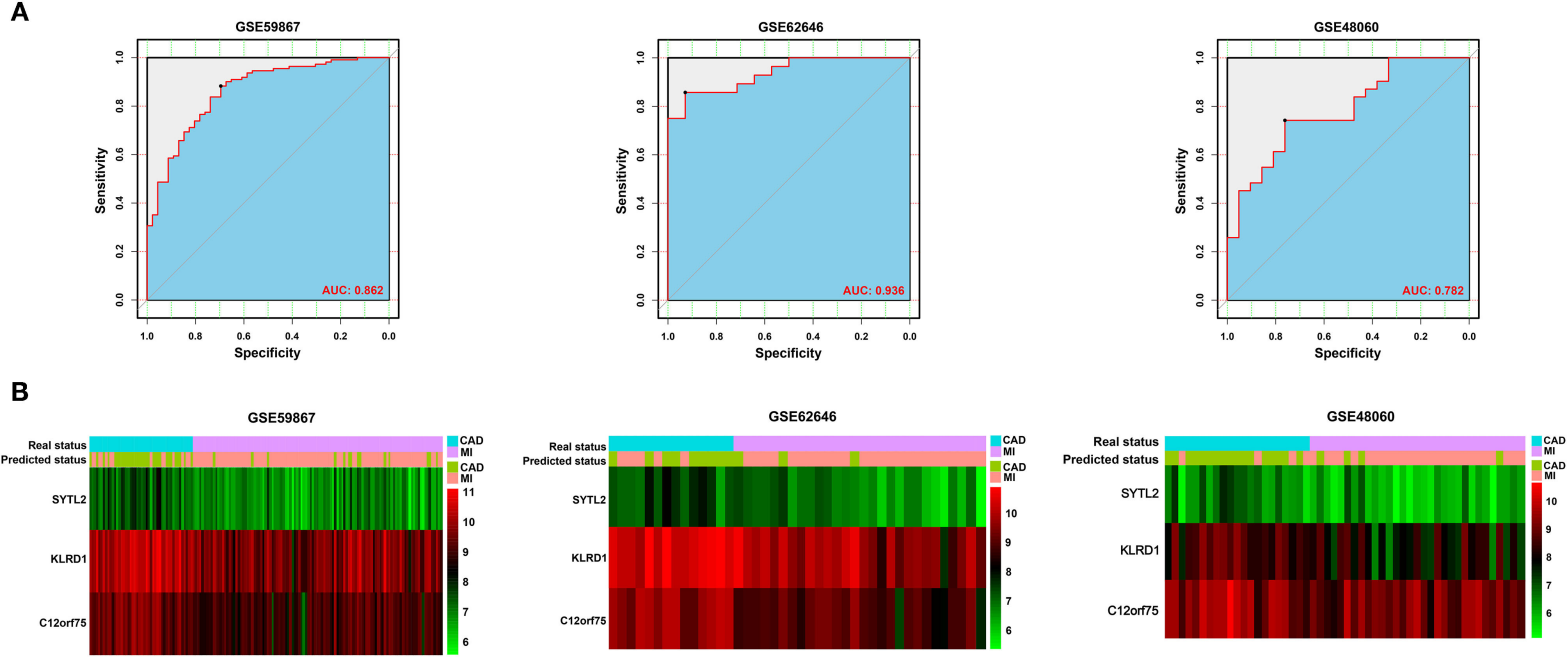
Validation of diagnostic biomarkers for SD-related MI. **(A)** ROC analysis revealed good diagnostic performance of the risk score associated with MI among the three datasets. **(B)** Unsupervised hierarchical clustering of risk scores in the diagnostic prediction model among the three MI-related datasets.

After constructing the model using LASSO regression analysis, the risk score was calculated for each sample based on the corresponding coefficients and expression value of genes as follows: Risk score = [(-0.02072662) × Expression value of SYTL2] + [(-0.13152628) × Expression value of KLRD1] + [(-0.06924624) × Expression value of C12orf75]. As shown in Figure 4A and Supplementary Figure 1, ROC analysis demonstrated favorable diagnostic efficacy of the above three biomarkers in discriminating MI from CAD samples with an AUC of 0.807 (95% CI 0.732–0.883) for SYTL2, 0.843 (95% CI 0.776–0.909) for KLRD1, and 0.829 (95% CI 0.758–0.901) for C12orf75. For the risk score, the diagnostic ability in terms of AUC was 0.862 (95% CI 0.800–0.924). The risk score also showed a high discrimination ability in the GSE62646 dataset with an AUC of 0.936 (95% CI 0.869–1.000). To externally validate the diagnostic value of the three identified biomarkers, the GSE48060 dataset was also used, and the results indicated a powerful diagnostic ability for the feature biomarkers (AUC 0.782, 95% CI 0.657–0.907). Unsupervised hierarchical clustering of three biomarkers showed different gene expression between MI and CAD samples with high sensitivity and specificity (Figure 4B).

### Enrichment of Immune Cells in the MI and CAD Samples

**Figure 5:**
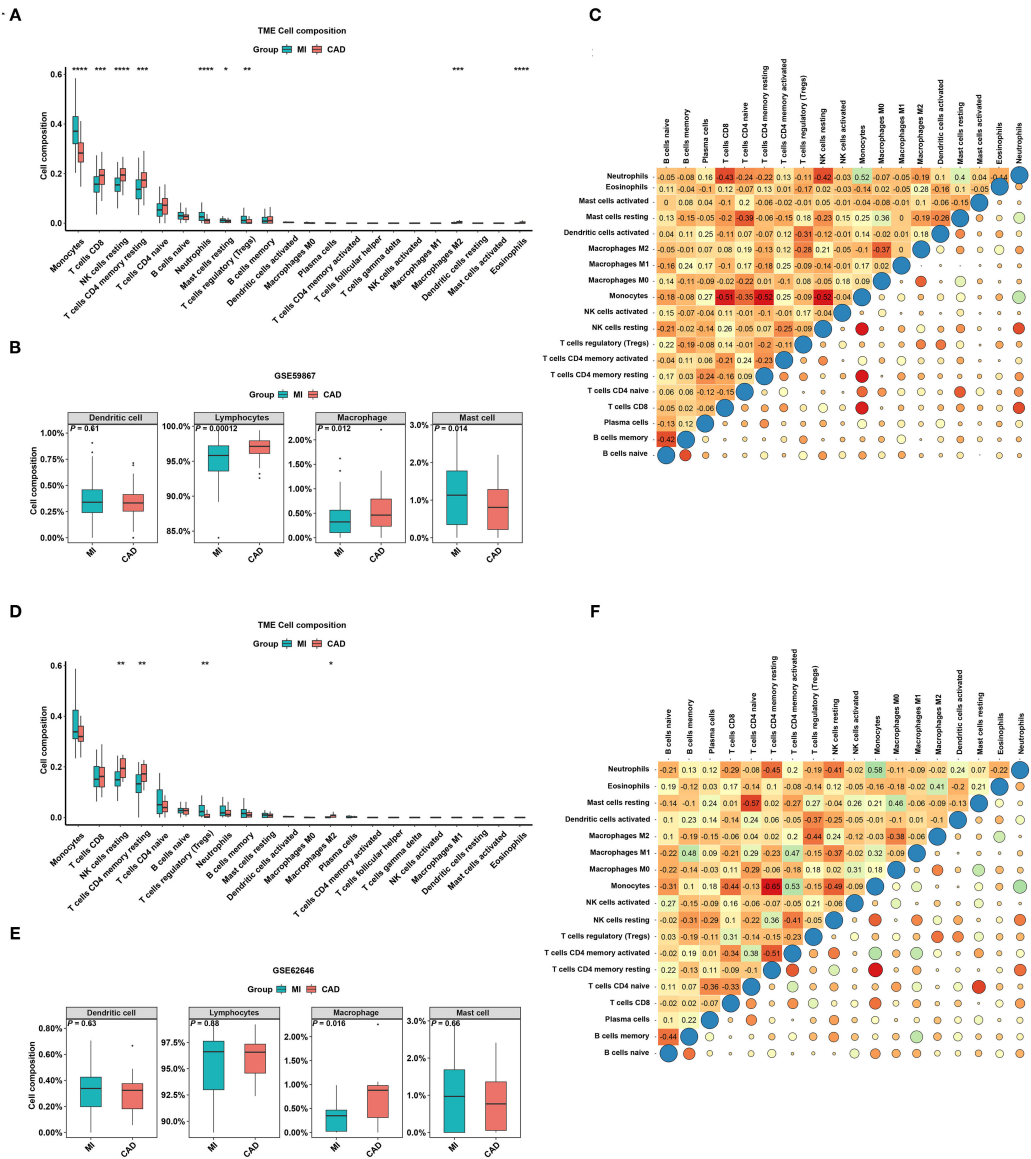
The distribution of 22 types of immune cells between the MI and CAD samples. **(A,D)** Violin plots of 22 types of immune cells that are differentially enriched in the **(A)** GSE59867 dataset and **(D)** GSE62646 dataset. **(B,E)** Violin plot of four aggregated immune cell types that are differentially enriched in the **(B)** GSE59867 dataset and **(E)** GSE62646 dataset. **(C,F)** Heatmap of correlations for 22 types of immune cells in the **(C)** GSE59867 dataset and **(F)** GSE62646 dataset. The size of the colored squares represents the strength of the correlation. Darker color implies stronger association. *P < 0.05; **P < 0.01; ***P < 0.001; ****P < 0.0001.

To better understand the difference in the enrichment degree of immune cell subtypes between the MI and CAD groups, 22 available immune cell subtypes, including the major cell types related to adaptive immunity [i.e., naïve B cells, memory B cells, naïve CD4 T cells, resting memory CD4 T cells, activated memory CD4 T cells, CD8 T cells, gamma delta T (Tgd) cells, T follicular helper (Tfh) cells, and regulatory T (Treg) cells] and innate immunity [i.e., activated dendritic cells (DCs), resting DCs, eosinophils, activated mast cells, macrophages (M0–M2), resting mast cells, monocytes, resting NK cells, activated natural killer (NK) cells, neutrophils, and plasma cells], were assessed using CIBERSORT. GSE59867 dataset results showed that five types of immune cells were significantly enriched in a higher proportion in the CAD group, and four types of immune cells were significantly increased in the MI samples (all *P* < 0.05) (Figure 5A). After clustering the 22 immune cell types into four aggregated immune cell types, the total lymphocytes and total macrophages were significantly enriched in the CAD samples, whereas mast cells were enriched in the MI group (all *P* < 0.05) (Figure 5B). The GSE62646 dataset was used to validate the distribution of immune cells. The proportions of three immune cell subtypes, including resting memory CD4 T cells, resting NK cells, and M2 macrophages, were significantly lower in MI samples compared with CAD samples (all *P* < 0.05) (Figure 5D). These results are consistent with the GSE59867 dataset results. However, only total macrophages were significantly enriched in the CAD groups (*P* < 0.05) (Figure 5E). As shown in Figures 5C,F, the interrelation among the various immune cell subtypes in the GSE59867 and GSE62646 datasets varied from weak to moderate.

### Immune Cell Infiltration in SD Samples and Control Samples

**Figure 6:**
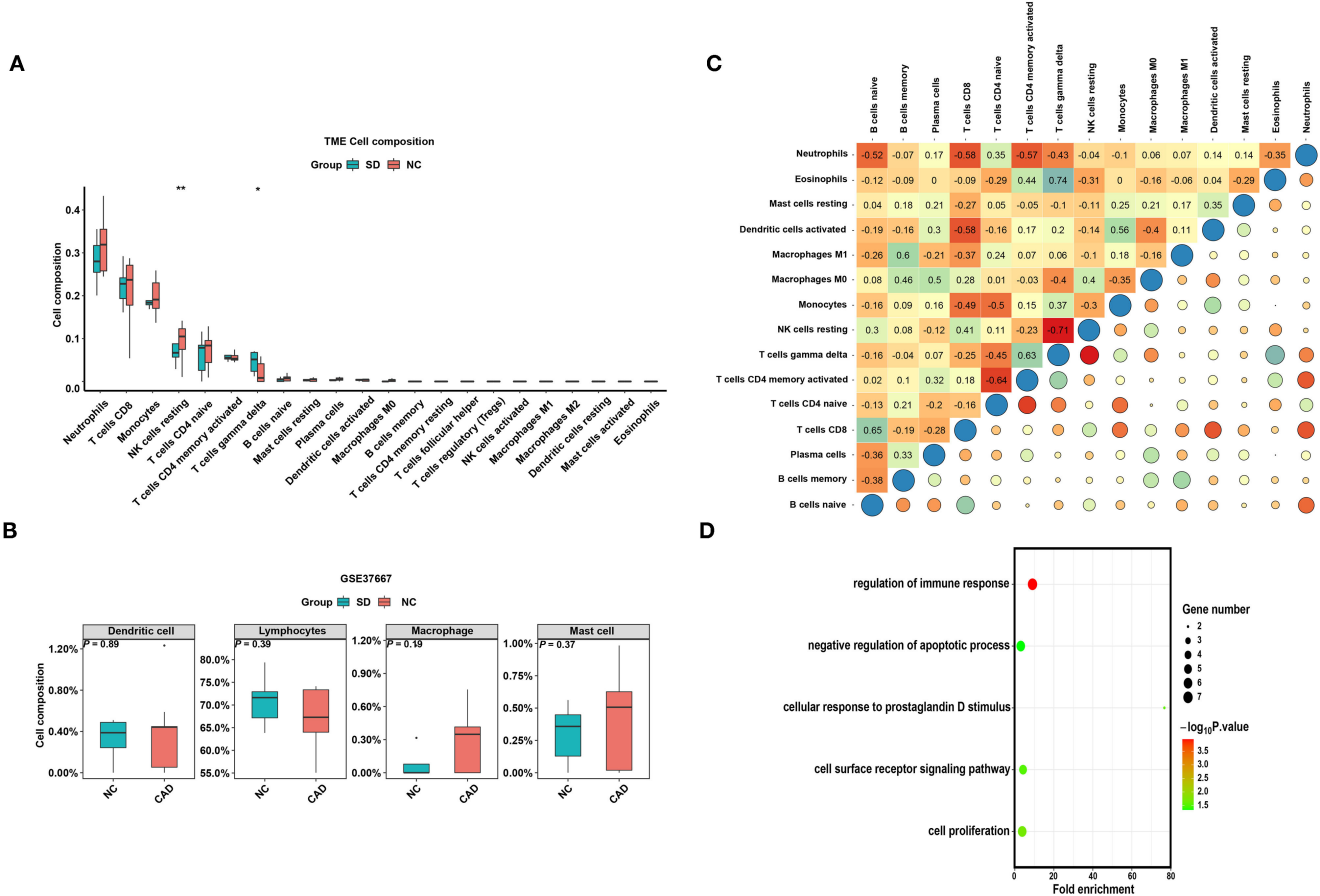
The distribution of 22 types of immune cells between the SD and control samples. **(A)** Violin plot of 22 types of immune cells differentially enriched between the SD and negative control (NC) samples in the GSE37667 dataset. **(B)** Violin plot of four aggregated immune cell types differentially enriched in the GSE37667 dataset. **(C)** Heatmap of correlation of 22 types of immune cells in the GSE37667 dataset. The size of the colored squares represents the strength of the correlation. Darker color implies stronger association. **(D)** Biological process of SD-DEGs from the GSE37667 dataset. *P < 0.05; **P < 0.01.

To verify whether the distribution of immune cells in the SD/control groups was consistent with that in the MI/CAD groups, we also employed the CIBERSORT algorithm on the GSE37667 dataset. The results indicated that the proportion of resting NK cells was significantly lower in the SD group than in the control group (*P* < 0.05) (Figure 6A). Although 4 aggregated immune cell types showed insignificant differences between the two groups, resting NK cells were negatively correlated with gamma delta T (Tgd) cells, which were significantly enriched in the SD samples (*P* < 0.05) (Figures 6B,C). These results suggested that immune cells, especially resting NK cells, were actively involved in SD-induced disease processes. To further analyse the functional enrichment during the SD process, GO enrichment analysis was performed with SD-DEGs using the online DAVID tool. As shown in Figure 6D, the SD-DEGs were significantly enriched in the following biological processes: regulation of immune response and negative regulation of apoptotic processes (all *P* < 0.05).

### Correlation of Diagnostic Biomarkers and Immune Cell Types

**Figure 7:**
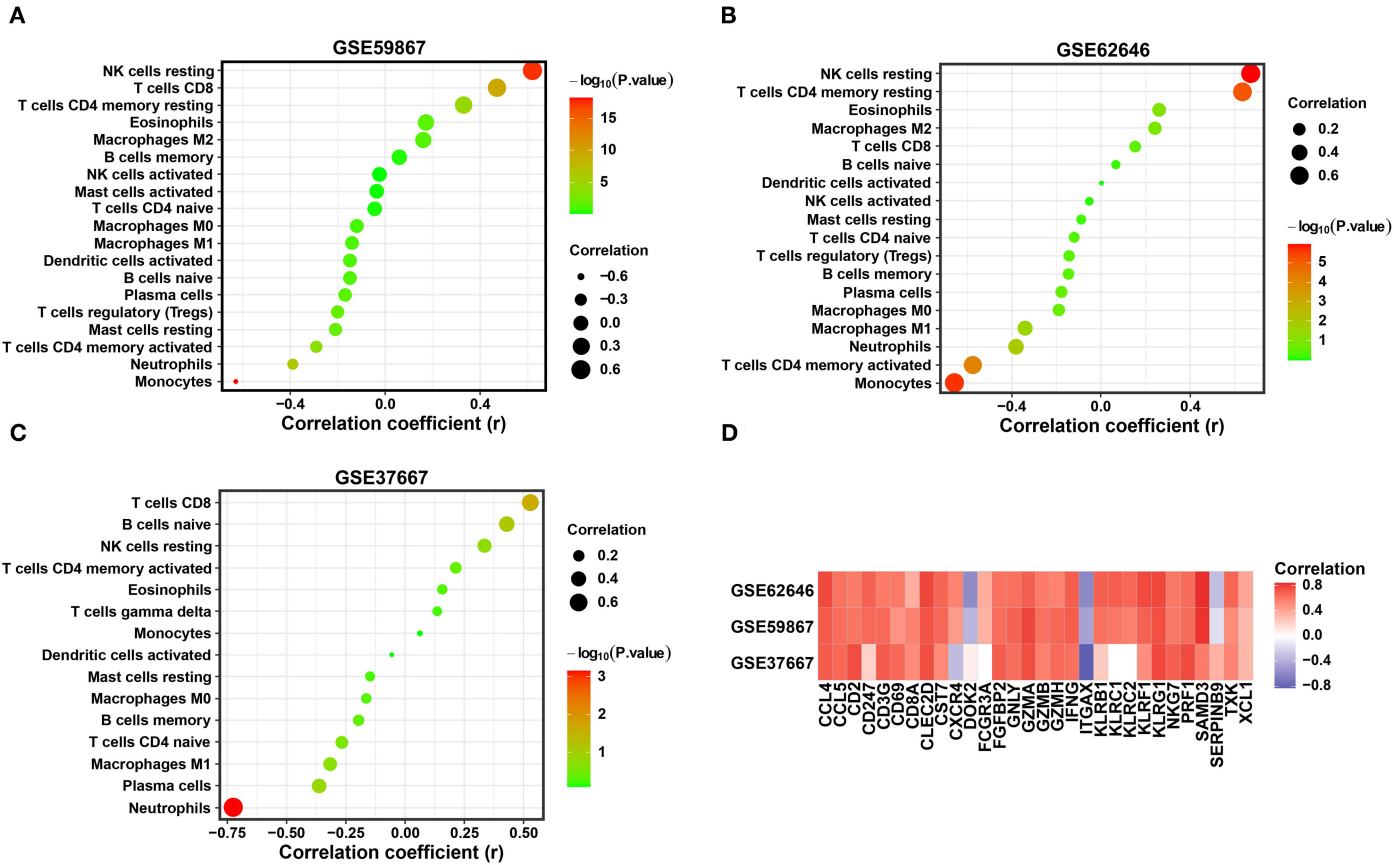
Correlation between the expression level of SYTL2 and enriched immune cells. **(A)** Correlation between the expression level of SYTL2 and enriched immune cells in the GSE59867 dataset. **(B)** Correlation between the expression level of SYTL2 and enriched immune cells in the GSE62646 dataset. **(C)** Correlation between the expression level of SYTL2 and enriched immune cells in the GSE37667 dataset. The size of the dots represents the strength of the correlation between SYTL2 and immune cells; the color of the dots represents the *P*-value; the greener the color, the larger the *P*-value; and the red the color, the lower the *P*-value. *P* < 0.05 was considered statistically significant. **(D)** Heatmap showing the correlation between SYTL2 and the marker genes of NK cells.

Combined with the above results, the expression levels of three diagnostic biomarkers were downregulated in both the SD and MI samples, and resting NK cells were not enriched in either the SD or MI groups among the three datasets. Next, we conducted correlation analyses in two MI-related datasets and an SD-related dataset to explore the relationship between the diagnostic biomarkers and immune cell types. As shown in Figures 7A–C, SYTL2 showed a significantly strong positive correlation with resting NK cells in the MI-related datasets as well as the SD dataset, which is consistent with the changes induced by MI or SD mentioned above (all *P* < 0.05). However, these trends were not reflected in KLRD1 and C12orf75 (Supplementary Figure 2). Moreover, we downloaded gene sets related to NK cell markers from CellMarker and performed Pearson correlation analysis between SYTL2 and gene sets in three datasets (Figure 7D). The results indicated that SYTL2 was also strongly positively correlated with most genes related to NK cell markers (all *P* < 0.05). These results indicate that SYTL2 plays an important role in regulating NK cell activation during the process of SD-induced MI.

### Involvement of SYTL2 in the NK Cell Signaling Pathway

**Figure 8:**
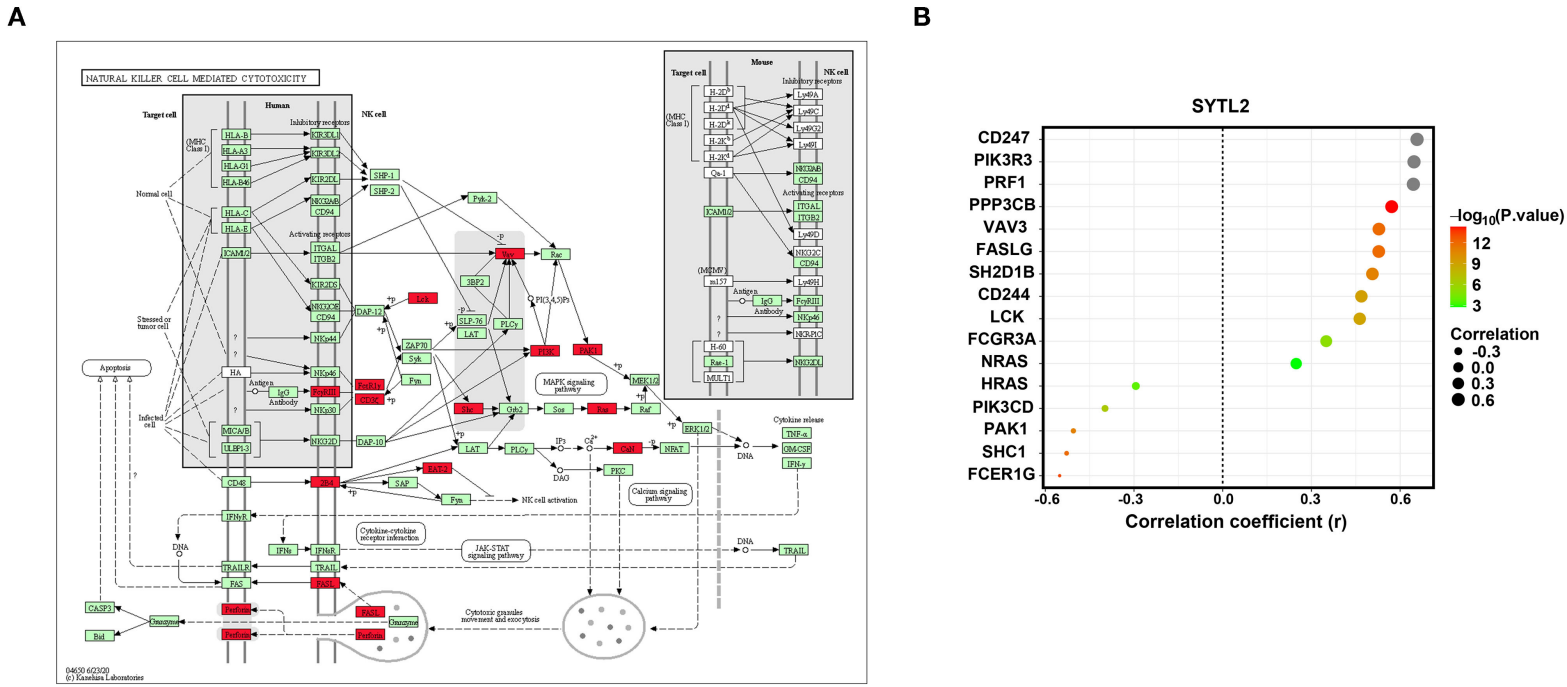
Association between SYTL2 and the NK cell-mediated cytotoxicity signaling pathway. **(A)** Regulation network of the NK cell-mediated cytotoxicity signaling pathway as obtained from the KEGG database. The red square indicates that the gene was significantly correlated with SYTL2 (*P* < 0.05). **(B)** Correlation between the expression level of SYTL2 and the genes in the NK cell-mediated cytotoxicity signaling pathway.

NK cell signaling pathways are of great importance for NK cell activation and are currently the target of several therapeutic strategies (25, 26). NK cells express many receptors that activate their cytotoxic and secretory functions, which contribute to immune defense (27). Therefore, we investigated the correlation of SYTL2 with the genes in the NK cell signaling pathway that were obtained from the KEGG database (Supplementary Table 2). As shown in Figure 8, SYTL2 was highly associated with the genes in the MAPK signaling pathway (PIK3R3, PIK3CD, SHC1, PAK1, NRAS, and HRAS, all *P*-values < 0.05), cytotoxic granule movement and exocytosis (PRF1 and FASLG, all *P*-values < 0.05), and NK cell activation (SH2D1B and CD244, all *P*-values < 0.05) and might be partially related to the calcium signaling pathway (PPP3CB, *P*-value < 0.05).

### Detection of Biological Function for SYTL2

**Figure 9:**
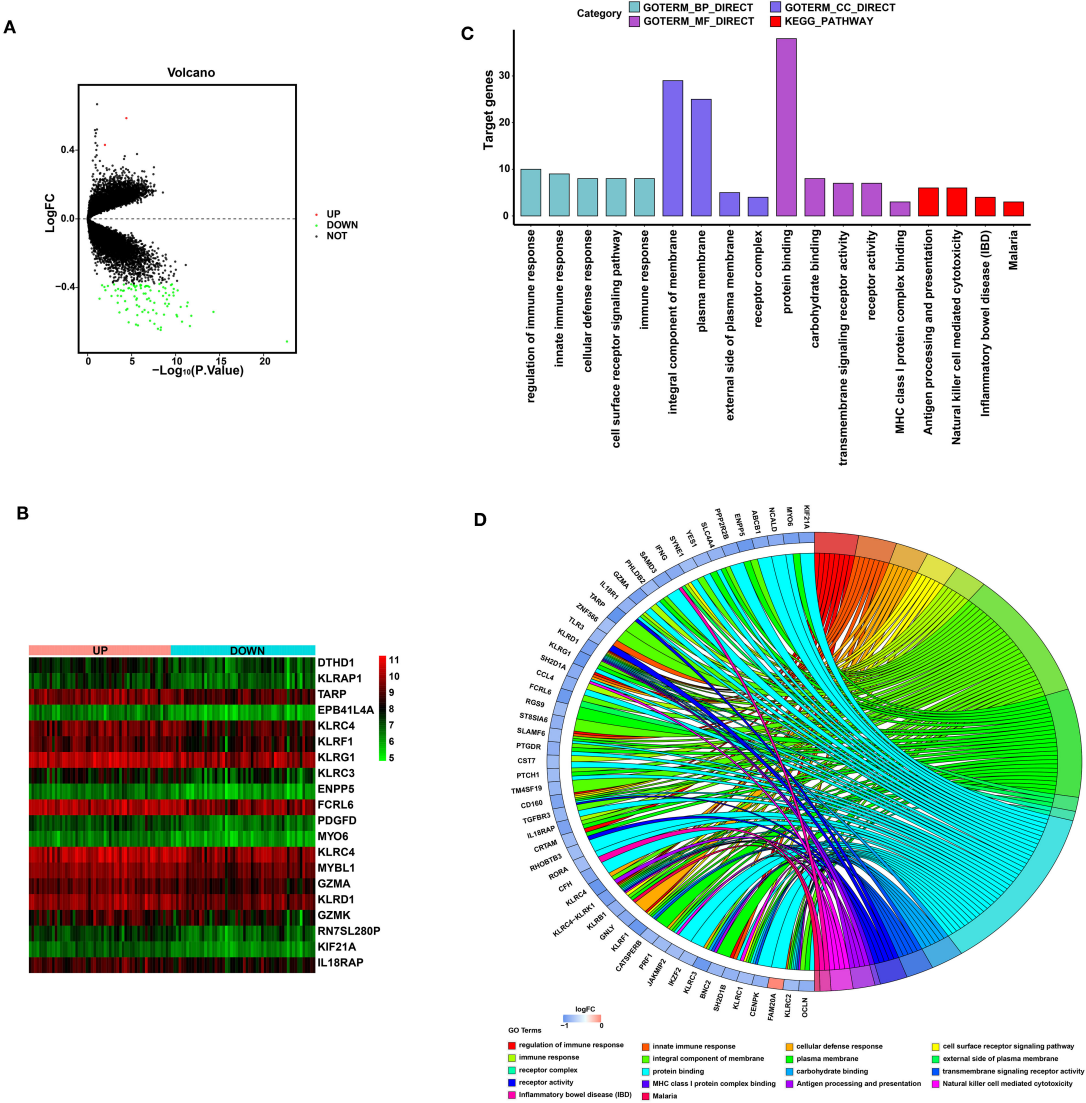
Significant pathways influenced by SYTL2 in the GSE59867 dataset. **(A)** Volcano plot of the GSE59867 dataset. A total of two upregulated DEGs and 91 downregulated DEGs were identified between low- and high-SYTL2 expression samples. **(B)** Heatmap of DEGs between low- and high-SYTL2 expression samples in the GSE59867 dataset. **(C)** DEGs with the top 15 enriched GO terms and KEGG terms. **(D)** Distribution of DEGs for different GO-enriched functions.

To explore the biological function of SYTL2 in the MI process, we obtained the DEGs using 111 MI samples from the GSE59867 dataset that were divided into the high-SYTL2 (*n* = 55) and the low-SYTL2 groups (*n* = 56) with the median value as the cut-off. A total of 2 upregulated DEGs and 91 downregulated DEGs were identified with the high-SYTL2 expression sample as a reference (Figures 9A,B). Then, GO and KEGG analyses were performed to analyse the DEGs, indicating the potential function of SYTL2. The results also suggested a strong association with NK cell-mediated cytotoxicity and regulation of the immune response (Figures 9C,D and Supplementary Table 3). Moreover, GSEA was performed between low- and high-SYTL2 expression samples in the GSE59867 dataset. Several immune pathways that involve SYTL2 in relation to MI, such as “natural killer cell mediated immunity,” “positive regulation of natural killer cell mediated cytotoxicity,” and “regulation of natural killer cell mediated immunity,” were identified (Supplementary Table 4).

## Discussion

MI is an extremely dangerous cardiovascular disease that causes rapid deterioration due to the sudden rupture of plaques associated with the chronic inflammatory disease called atherosclerosis. Sleep is a key factor in regulating immune homeostasis of the body. The imbalance of circulating immune cells caused by sleep deprivation may represent a risk factor leading to the rapid deterioration of plaques. Therefore, it is vital to explore diagnostic biomarkers and analyse the association with immune cell enrichment to improve the prognosis of MI.

In the present study, we identified 1,599 DEGs between the MI and CAD PBMC samples based on the GSE59867 and GSE62646 datasets as well as 102 DEGs between SD and control PBMC samples from the GSE37667 dataset. Then, a total of 10 co-DEGs were obtained from the MI-DEGs and SD-DEGs. After applying LASSO regression analysis, SYTL2, KLRD1, and C12orf75 were selected and validated as diagnostic biomarkers using ROC analysis. Next, we found that resting NK cells were downregulated in both the MI samples and SD samples, which is similar to the change noted for SYTL2. Importantly, SYTL2 was strongly positively correlated not only with resting NK cells but also with most genes related to NK cell markers among the MI datasets and SD dataset. Moreover, we performed correlation analysis between SYTL2 and the genes in the NK cell signaling pathway that were obtained from the KEGG database. The results indicated that SYTL2 was highly associated with genes in the MAPK signaling pathway, cytotoxic granule movement and exocytosis, and NK cell activation. Furthermore, GSEA and KEGG analyses suggested a strong association with regulation of the immune response and NK cell-mediated cytotoxicity for the DEGs between low- and high-SYTL2 expression samples in GSE59867, which were classified based on a median cut-off value. All of the above evidence demonstrated that the biological function of SYTL2 might be strongly correlated with the immune response in SD-related MI processes, especially with NK cells.

Biomarkers in the circulation play a key role in risk stratification and therapeutic management of cardiovascular diseases due to the difficulty of obtaining anatomical tissue biopsies (28, 29). A previous study indicated that the expression levels of over 80% of peripheral blood transcriptomes were shared among 9 different human tissue types, suggesting that PBMCs are sensitive to ongoing cardiac dysfunction and respond by altering their transcriptome. However, the reason why tissue-specific upregulated or downregulated gene patterns are synchronized to circulating cells remains unclear (18, 30). In this context, our current study highlights the potential to use mRNA signatures in PBMCs as diagnostic biomarkers of SD-related MI. PBMCs are an innate circulating cell population with inflammatory properties that have significant associations with atherosclerotic plaque formation and MI risk and progression (31–33). A transcriptome study of PBMCs from early onset MI patients indicated that lncRNA-*NEAT1* expression levels were significantly downregulated in MI PBMC samples. LncRNA-*NEAT1* was identified as an immunoregulator affecting T cell and monocyte-macrophage lineage differentiation and functions *in vivo*, which may impact the course of diseases (34). ALK4 expression levels in PBMCs of MI patients were significantly higher than that in PBMCs of healthy volunteers, and ALK4 function was associated with cardiac inflammation and vulnerability to ventricular arrhythmia after acute myocardial injury. The establishment of an MI mouse model suggested the potential involvement of macrophage-mediated ALK4 expression in the inflammatory phase of MI (35). Moreover, a bioinformatics study based on the gene expression profiles of PBMCs between AMI samples and controls was performed. IL1R2, IRAK3, and THBD were collectively identified as a diagnostic marker of AMI and showed a close correlation with immune cells, such as M2 macrophages, monocytes, activated NK cells, and gamma delta T cells (14). The present study identified SYTL2, KLRD1, and C12orf75 as diagnostic markers of SD-related MI and demonstrated that immune cells, especially resting NK cells, played important roles in disease progression. Therefore, this gene set represents a promising measurement from a clinical perspective. Specifically, the PBMC expression profile of atherosclerosis patients who undergo SD might have prognostic value regarding the clinical course or response to anti-inflammatory treatment.

The amount of sleep time has drastically decreased in modern society due to changes in lifestyle behavior and the presence of sleep disorders. Several intermediate pathophysiological mechanisms have been reported to be induced by short sleep duration, such as inflammatory responses, atherosclerosis, oxidative stress, and insulin resistance, resulting in the development of cardiovascular and metabolic disorders (36–40). In a large prospective study from London with more than 10,000 participants and a mean follow-up time of 15 years, participants with a short sleep duration showed the highest risk of CAD (MI and angina, relative risk 1.55, 95% CI 1.33–1.81), especially in people with sleep disorders (41). Sleep loss exerts a strong regulatory influence on peripheral levels of inflammatory mediators of the immune response, which contributes to the development of atherosclerosis (42). Inflammatory cytokines, such as IL-1α, IL-1β, IL-6, and TNF-α, exhibit a positive linear association with habitual short sleep duration. C-reactive protein (CRP), which is considered a predictor of cardiovascular events, was also elevated in plasma after partial SD, and the levels remained high even after two nights of sleep recovery (42, 43). As the main source of these cytokines, the expression levels in monocytes are strongly regulated by circadian rhythms (44). Generally, the leukocyte population increases after acute SD. However, the number of circulating NK cells was decreased after SD, which subsequently led to an increase in B and T lymphocytes and total white blood cells (43). A previous study indicated that the number of apoptotic NK cells in peripheral blood was significantly increased in CAD patients compared to healthy patients, and this effect was induced by oxidative stress (45). Our study found that resting NK cells were enriched in neither MI samples nor SD samples. One reasonable hypothesis is that NK cells undergo apoptosis due to oxidative stress induced by SD, which subsequently stimulates the increase in other inflammatory cells and enhances the inflammatory response, finally increasing cardiovascular events.

Three diagnostic biomarkers, including SYTL2, KLRD1, and C12orf75, were identified to be associated with SD-related MI. The synaptotagmin-like protein homology domain (SHD) of SYTL2 specifically binds to the GTP-bound form of Ras-related protein Rab-27A (RAB27A), which suggests a role of vesicle trafficking and exocytosis in epithelial cells and haematopoietic cells, including neutrophils, cytotoxic T cells, NK cells, and mast cells (46–48). SYTL2 has been reported to control the podocalyxin-rich vesicles tethering and fusion in conjunction with Rab27/Rab3/Rab8 *via* synaptotagmin-like protein 4a (Slp4a) to promote vascular lumen formation (49). SYTL2 is recruited to the apical membrane where it regulates secretion of Weibel-Palade Body components into the luminal space (50). Knockout of SYTL2 blunts the vascular lumen formation during angiogenic development, suggested its potential role in the setting of MI (50). KLRD1, which is also named CD94, is an antigen preferentially expressed on NK cells (51). NK cells are important in the onset of AMI given their ability to secrete IFN-γ and other inflammatory cytokines (52). However, NK cell activity and quantity were suppressed in MI patients with significant mRNA downregulation of inhibitory and activating NK cell receptors (53, 54). The specific mechanism of these clinical manifestations remains unclear. The present study might provide potential evidence that the downregulated expression levels of SYTL2 and KLRD1 in SD-related MI patients lead to dysfunction of exocytosis in NK cells and suppress NK cell activity, which subsequently contributes to neutrophil and T cell activity and the immune response. This hypothesis also needs to be further validated by *in vivo* and *in vitro* experiments.

It should be noted that cardiac troponin (cTn), including cardiac-specific troponin T (cTnT) and I (cTnI), are now widely used as a gold standard to identify patients with MI (55). Despite the cardiac specificity of troponin, there are other clinical conditions except for MI in which troponin may be elevated, including cardiac and non-cardiac causes (56). Previous studies indicated that high-sensitivity cTnI was elevated in patients with obstructive sleep apnea (OSA), which higher OSA severity was related to higher concentrations of high-sensitivity cTnI (57, 58). However, the presence of OSA may have a protective effect on myocardial ischemic injury in the setting of AMI, which was manifested as lower concentrations of cTnI than patients without OSA (59). These above evidences showed that the use of troponin to diagnose MI caused by sleep disorders is still controversial. Thus, the exploration for other interesting insights of areas like peripheral blood genomics biomarkers might assist troponin in the diagnosis of true MI in patients with sleep disorder.

Several unavoidable limitations in the present study should be acknowledged. First, the sample size of the included study was relatively small, and only 9 individuals were recruited in the GSE37667 dataset for SD. Second, due to the rapid progress of sequencing technology, heterogeneity exists between different batches and experimental platforms. Third, the study was performed based on microarray datasets with two different populations to explore the role of genes from PBMCs in SD-related MI. However, direct evidence, such as clinical studies or *in vivo* experiments, are not available to support the conclusion. It is better to design study as in the previous literature. They collected peripheral blood samples from 302 patients for a case-control study to further confirm the bioinformatics analysis results based on the GSE59867 and GSE62646 datasets that dysregulated circulating hub genes expression were associated with MI development (60). Therefore, experiments using *in vitro* and *in vivo* models as well as prospective clinical studies will be indispensable for validation of the diagnostic and theragnostic value of these biomarkers.

## Conclusion

In summary, a set of genes from PBMCs, including SYTL2, KLRD1, and C12orf75, were identified as diagnostic biomarkers for SD-related MI. SYTL2 exhibited a strong positive correlation with resting NK cells, which were both downregulated in the MI samples and SD samples and involved in NK cell signaling pathways, including the MAPK signaling pathway, cytotoxic granule movement and exocytosis, and NK cell activation. These diagnostic biomarkers and hypothetical signaling axes may provide prognostic value and therapeutic targets for SD-related MI.

## Data Availability Statement

The datasets presented in this study can be found in online repositories. The names of the repository/repositories and accession number(s) can be found in the article/Supplementary Material.

## Author Contributions

EW supervised the project, designed the study, interpreted the data, and wrote and reviewed the manuscript. XC and QL performed data management and analyzed the data. ZZ and MY took part in analyzing the data. XC wrote the first draft of the manuscript. All authors approved the final version of the manuscript.

## Funding

This study was funded by the National Key Research and Development Program of China (No: 2020YFC2005300).

## Conflict of Interest

The authors declare that the research was conducted in the absence of any commercial or financial relationships that could be construed as a potential conflict of interest.

## Publisher's Note

All claims expressed in this article are solely those of the authors and do not necessarily represent those of their affiliated organizations, or those of the publisher, the editors and the reviewers. Any product that may be evaluated in this article, or claim that may be made by its manufacturer, is not guaranteed or endorsed by the publisher.

## Supplementary Material

Supplementary Figure 1 Validation of diagnostic biomarkers for SD-related MI.

Supplementary Figure 2 Correlation between the expression level of two diagnostic biomarkers and immune cell enrichment.
