# Supplementary figures and images for: Identification of Potential Diagnostic Biomarkers From Circulating Cells During the Course of Sleep Deprivation-Related Myocardial Infarction Based on Bioinformatics Analyses
Source: Front Cardiovasc Med. 2022 Mar 17;9:843426. doi: 10.3389/fcvm.2022.843426 (PMC8969017; doi:10.3389/fcvm.2022.843426)

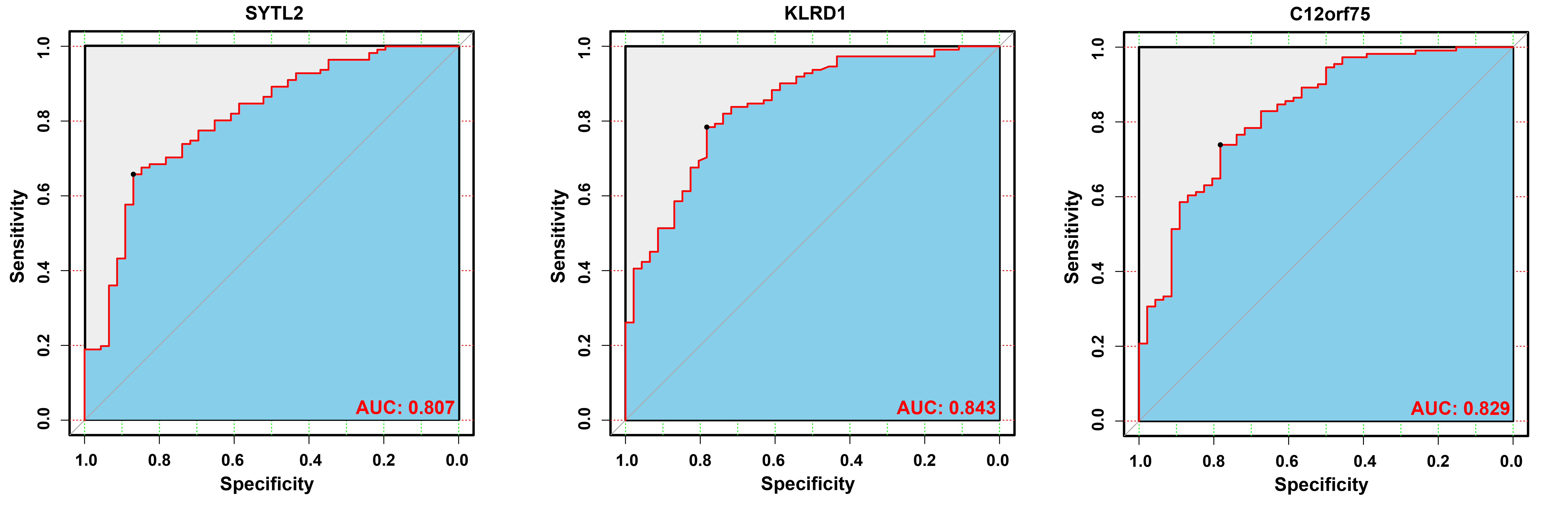

Supplement: Supplementary Figure 1 — Validation of diagnostic biomarkers for SD-related MI. [file Image_1.TIF]

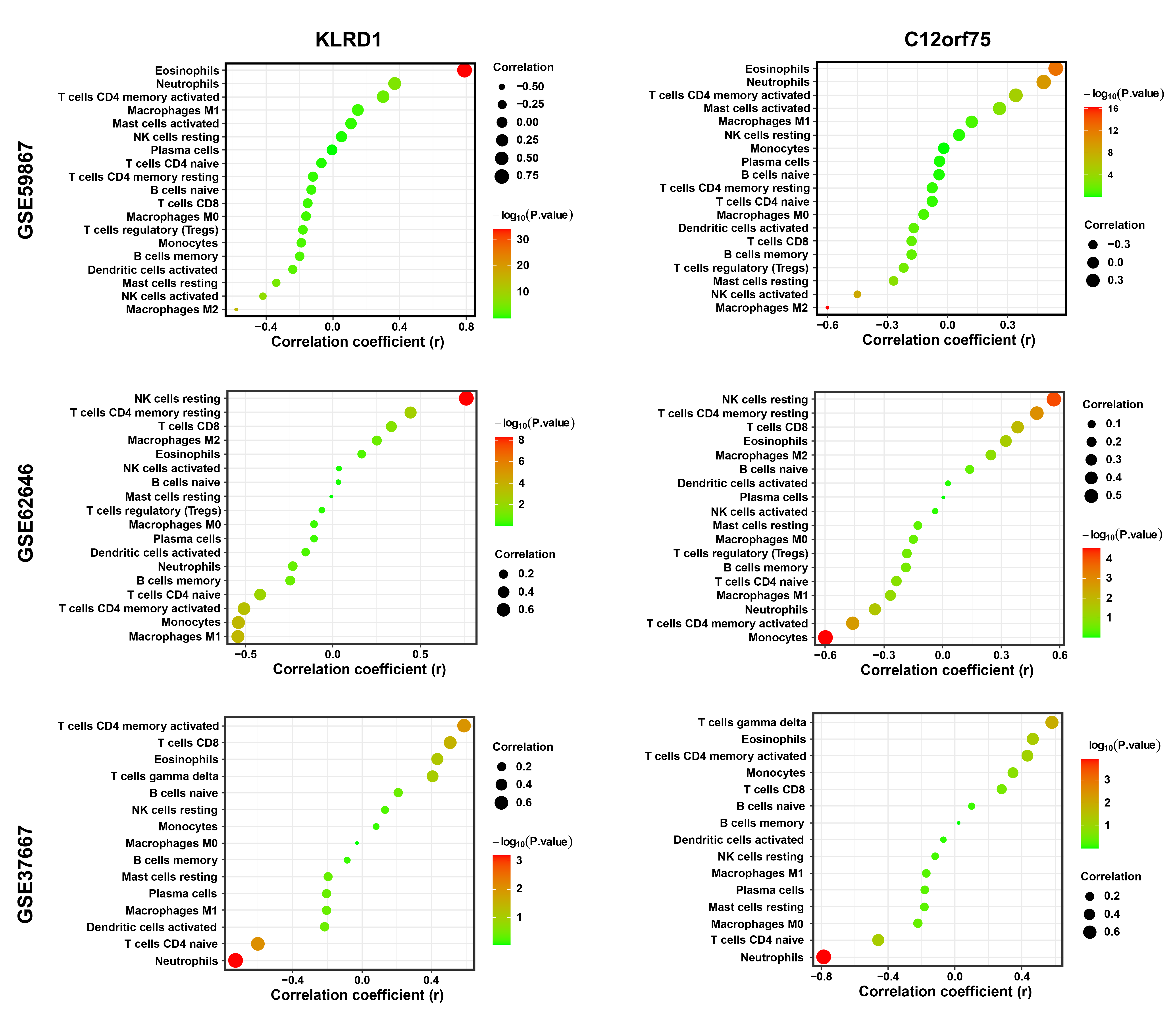

Supplement: Supplementary Figure 2 — Correlation between the expression level of two diagnostic biomarkers and immune cell enrichment. [file Image_2.TIF]
